# Astragalus polysaccharide antagonized anti PD-1 efficacy in melanoma through inhibiting JAK-STAT1/CIITA and TLR/NF-κB signaling

**DOI:** 10.3389/fimmu.2026.1947786

**Published:** 2026-09-14

**Authors:** Ruilong Wang, Qiao Li, Mengguo Liu, Jinshu Yu, Min Peng, Xiaohong Deng, Rui Lei, Xiao Liu, Zhihao Li, Hiochon Leong, Jie Ren, Jinfeng Wu

**Affiliations:** 1 Department of Dermatology, Huashan Hospital, Fudan University, Shanghai, China; 2 Department of Dermatology, Nantong Haimen People’s Hospital, Nantong, China; 3 Department of Dermatology, Chuxiong Autonomous Prefecture Hospital of Traditional Chinese Medicine, Chuxiong, China; 4 Department of Integrative Medicine, Huashan Hospital, Fudan University, Shanghai, China; 5 Suzhou Medical College of Soochow University, Suzhou, China

**Keywords:** anti PD-1, astragalus polysaccharide, JAK-STAT1, melanoma, TLR

## Abstract

**Objective:**

Advanced melanoma remains a major clinical challenge due to resistance to immune checkpoint inhibitors (ICIs, e.g., anti PD-1). Astragalus Polysaccharide (APS) is a clinically approved immunomodulator for cancer treatment. However, its interaction with anti PD-1 remains largely unknown. This study aims to explore the efficacy and underlying mechanism of APS combined with anti PD-1 in melanoma.

**Methods:**

For *in vivo* studies, the anti-tumor effect of APS combined with anti PD-1 was evaluated in a B16-OVA melanoma mouse model. For mechanism exploration, immunohistochemistry, flow cytometry, ELISA, PCR array, qRT-PCR, Western blotting and confocal microscopy were employed to detect tumor immune microenvironment changes and related signaling pathway activation.

**Results:**

Unexpectedly, APS significantly antagonized the anti-tumor efficacy of anti PD-1 in melanoma mice. APS reversed anti PD-1-induced infiltration of CD4^+^ T cells, CD8^+^ T cells and dendritic cells (DCs) in the tumor microenvironment, and impaired DC maturation and CD4^+^ T cell activation. Mechanistically, APS significantly inhibited JAK-STAT1 signaling, downregulated MHCII master regulator CIITA, and subsequently reduced MHCII expression. Meanwhile, APS markedly suppressed TLR signaling and attenuated NF-κB mediated inflammatory responses in B16-OVA melanoma cells and DCs.

**Conclusion:**

APS antagonized anti PD-1 efficacy by inducing an immunosuppressive microenvironment via dual inhibition of JAK-STAT1/CIITA signaling and TLR/NF-κB signaling. This study suggests that the combination of natural polysaccharides and ICIs may warrant caution, although further studies are needed to validate these findings and determine their clinical relevance.

## 1 Introduction

Cutaneous melanoma is the deadliest form of skin cancer (1). Immune checkpoint inhibitors (ICIs, e.g., anti PD-1) have revolutionized the treatment landscape for advanced melanoma, significantly improved patient survival rates (2, 3). However, resistance to anti PD-1 remains a major clinical challenge, with approximately 30%-40% of patients ultimately experiencing treatment failure (3). Enhancing the sensitivity of anti PD-1 is therefore a major research focus.**​**

Major histocompatibility complex class II (MHCII) molecules play critical role in activating CD4^+^ T-cells and indirectly amplifying CD8^+^ T-cell responses (4). The traditional view holds that specialized antigen-presenting cells (APCs), such as DCs, are capable of expressing MHCII molecules (5, 6). However, tumor cells including melanoma cells have been found to express tumor-specific MHCII (tsMHCII), which shows a stronger correlation with treatment response and prognosis in patients receiving anti PD-1 therapy (7). MHCII transcription is governed by the class II transactivator (CIITA), which acts as its master regulator (8).CIITA itself is tightly controlled by the JAK-STAT pathway (9). Within the tumour microenvironment, functional JAK-STAT1 signaling is indispensable for IFN-γ-driven CIITA transcription and sufficient MHC-II expression, and defects in this signaling cascade commonly drive tumour immune evasion (10). Notably, the JAK–STAT pathway critically contributes to the efficacy of anti PD-1 therapy (11).

Toll-like receptor (TLR) pathway, a cornerstone of innate immunity, initiates signaling cascades upon recognition of pathogen-associated molecular patterns (PAMPs), primarily via activating NF-κB signaling and inducing type I interferons (12). This drives pro-inflammatory cytokines production (e.g., TNF-α, IL-6, and IL-12) and dendritic cells (DCs) maturation (12). It has been reported that TLR agonists functioned as potent sensitizers of ICIs by reprogramming the tumor microenvironment and eliciting both innate and adaptive immune responses (13).

Natural polysaccharides are high molecular weight biopolymers consisting of numerous monosaccharide units linked together by glycosidic bonds (14). These macromolecules are widely distributed across diverse biological sources, including plants, animals, fungi, algae, and microorganisms (14). Many polysaccharides have been developed into clinically approved drugs, such as Astragalus polysaccharide (APS), Lentinan, and Poria cocos polysaccharide. These polysaccharides exhibit a broad spectrum of biological activities, such as immunomodulatory effects, antitumor properties, antioxidant activity, and beneficial roles in maintaining gut health (15). APS, a major bioactive component derived from the Qi-tonifying Chinese herb Astragalus, has been shown to improve the immunity of cancer patients (16). In this study, we investigated the efficacy of APS combined with anti PD-1 on melanoma and determined the mechanisms through JAK-STAT1/CIITA signaling and TLR/NF-κB signaling.

## 2 Methods

### 2.1 Reagents

APS (Cat# HY-159066) was purchased from MCE (Shanghai, China). Anti-mouse PD-1 (CD279) was obtained from Bioxcell (Lebanon, NH, USA). Anti-mouse antibodies for flow cytometry were acquired from Biolegend (San Diego, CA, USA): MHC II-BV605 (Cat#107639), CD69-PE (Cat#104508), CD80-BV421 (Cat#104726), CD86-APC (Cat#105012), and CD11c-PE/Cy7 (Cat#117318). Anti-mouse antibodies for immunohistochemistry were purchased from Servicebio (Wuhan, China): CD4 (Cat#GB15064), CD8 (Cat#GB15068), and CD11c (Cat#GB11059). NF-κB (Cat#T55034S)) was supplied by Abmart (Berkeley Heights, NJ, USA), Phospho-STAT1 (Cat#7649T) was obtained from Cell Signaling (Danvers, MA, USA), STAT1 (Cat#10144-2-AP) and β-actin (Cat#66009) were purchased from Proteintech (Wuhan, China). CIITA (Cat#5979) was supplied by ProSci (Fort Collins, CO, USA).

### 2.2 Cell lines and animals

The B16-OVA murine melanoma cell line was obtained from the Cell Bank of the Chinese Academy of Sciences (Shanghai, China) and cultured in RPMI 1640 medium supplemented with 10% fetal bovine serum (FBS, ScienCell, Carsbad, CA, USA), under standard cell culture conditions (37 °C, 5% CO_2_). Female C57BL/6J mice, aged 6 weeks, were sourced from Vital River Laboratory Animal Technology (Zhejiang, China) and housed in the specific pathogen-free facility of Fudan University. The mice were maintained at a constant temperature (22 ± 1 °C) and humidity (60% ± 2%) with a 12-hour light/dark cycle, and had free access to food and water. All animal experiments were approved by the Institutional Animal Care and Use Committee of Fudan University (202012035S).

### 2.3 Animal experiments

To evaluate the therapeutic effects of APS, anti PD-1, and their combination, C57BL/6J mice bearing B16-OVA tumors were used. A total of 1×10^6^ B16-OVA cells suspended in 100 μL Phosphate Buffered Saline (PBS) were subcutaneously inoculated into the right flank of C57BL/6J mice. Once the tumors reached a size of approximately 40–80 mm (3), the mice were randomly assigned to four groups (n=6 per group): control group (saline, intraperitoneal injection (i.p.) daily), anti PD-1 group (i.p.) (10 mg/kg every three days for three times), APS group (i.p.) (200 mg/kg, daily), and APS combined with anti PD-1 group (i.p.). The 200 mg/kg/day APS dose is a widely validated effective dose for *in vivo* immunomodulatory and anti-tumor studies (17–19). Comparable regimens using 10 mg/kg anti PD-1 at approximately 3-day intervals have been extensively employed in B16-OVA melanoma models (20, 21). Sample size (6 mice per group) was chosen based on prior B16-OVA/anti-PD-1 longitudinal tumor-growth studies (22, 23). Tumor size was measured every 2 days using digital calipers (three replicates per time point). Tumor volume was calculated using the formula ab (2)/2 (a = longest diameter, b = shortest diameter). During the drug administration period, mice that died spontaneously or had a tumor volume exceeding 2000 mm3 were excluded from subsequent statistical analysis. Mice were euthanized at day 18, or when tumors became necrotic, or tumor volume exceeded 2000 mm³. Euthanasia was performed by gradual CO₂ inhalation (30% chamber volume/min displacement rate), followed by cervical dislocation to ensure death before tumor tissue collection.

### 2.4 Immunohistochemistry

Immunohistochemistry (IHC) was conducted following previously established protocols. In brief, tumor tissues were fixed in 4% paraformaldehyde, embedded in paraffin, and sectioned into slices of 8-10 μm. The sections were incubated overnight at 4 °C with primary antibodies against CD4, CD8, CD11c, and MHCII, followed by a 1-hour incubation at room temperature with secondary antibodies in a 1% BSA solution. Representative images were captured using an Olympus IX83 microscope.

### 2.5 Network pharmacological analysis

First, potential targets of APS were identified using the Genecards database, with additional targets related to “melanoma” and “anti PD-1” extracted. Overlapping genes were determined using a Venn diagram generated via Draw Venn Diagram (https://www.xiantaozi.com). Subsequently, protein-protein interaction (PPI) analysis was performed on genes with a confidence score >0.9 in the STRING database (https://cn.string-db.org), and 31 core targets were selected using the MCC algorithm in Cytoscape 3.8.2 with the cytoHubba plugin. Finally, GO enrichment and KEGG pathway enrichment analyses of the overlapping targets were conducted using the DAVID database (https://davidbioinformatics.nih.gov/), with the top 12 GO terms and 17 key signaling pathways visualized using bioinformatics tools.

### 2.6 PCR array

Gene expression profiles were evaluated utilizing a mouse Toll-Like Receptor Signaling Pathway PCR Array plate, following the instructions provided by the manufacturer (Wcgene Biotech, Shanghai, China). The data were subsequently analyzed using Wcgene Biotech software. Data were normalized to the reference gene based on cycle threshold (Ct) values. log_2_ (fold change) was calculated based on the 2^−ΔΔCt^ method.

### 2.7 qRT-PCR, ELISA and western blot

Experiments were conducted according to our previous study (24). For qRT-PCR analysis, total RNA was extracted using TRIzol reagent and reverse-transcribed into cDNA. Quantitative PCR was performed using SYBR Green Master Mix, and relative gene expression was calculated using the 2−ΔΔCt method. ELISA assays were conducted according to the manufacturers’ instructions. Western blotting was performed using standard procedures including protein extraction, SDS-PAGE separation, PVDF membrane transfer, antibody incubation, and chemiluminescent detection. The primers utilized in this study were listed below:

Mouse- *Gapdh*-F, AGGTCGGTGTGAACGGATTTG.
Mouse- *Gapdh*-R, TGTAGACCATGTAGTTGAGGTCA.
Mouse- *Ciita*-F, CTACTTCAGGCAGCAGAGGAGA.
Mouse- Ciita-R, GCTGTGTCTTCCGAGGAACTTC.
Mouse- *H2Aa*-F, TCAGTCGCAGACGGTGTTTAT.
Mouse- *H2Aa*-R, GGGGGCTGGAATCTCAGGT.
Mouse- *H2Ab1*-F, AGCCCCATCACTGTGGAGT.
Mouse- *H2Ab1*-R, GATGCCGCTCAACATCTTGC.
Mouse- *Tnf*-F, TGCCTCCTCTTTTGCTTATGTT.
Mouse- *Tnf*-R, AGGTTCAGTGATGTAGCGACAG.
Mouse- *Il6*-F, TAGTCCTTCCTACCCCAATTTCC.
Mouse- *Il6*-R, TTGGTCCTTAGCCACTCCTTC.

### 2.8 Confocal microscopy

B16-OVA cells were seeded onto the confocal dishes at a density of 3 × 10⁴ cells per dish and allowed to reach full confluence. Cells were pretreated with APS (200μg/mL) for 2h, then treated with lipopolysaccharide (LPS, 200ng/mL) and IFN-γ (10 ng/mL) for 48h. After that, the cells were fixed and blocked. Subsequently, cells were incubated with a NF-κB antibody at 4 °C overnight in the dark. After washing with PBS, samples were incubated with fluorophore-conjugated secondary anti-bodies for 1h at room temperature, cells were then subjected to staining with 4′, 6-diamidino-2-phenylindole (DAPI) in darkness for 10 min. Imaging was performed using a laser scanning confocal microscope (Leica TCS SP8). Data were analyzed using NIS-Elements Viewer 5.22.

### 2.9 Generation and culture of bone marrow-derived dendritic cells

Bone marrow cells were flushed from the femurs and tibias of 6-8-week-old female C57BL/6J mice using RPMI 1640 medium. After incubating with red blood cells lysis buffer, the cells were washed with PBS and resuspended in RPMI 1640 medium containing 10% FBS, 0.1% streptomycin, 100 U/mL penicillin, 40 ng/mL macrophage colony-stimulating factor (mGM-CSF), and 20 ng/mL interleukin-4 (IL-4). The culture medium was replaced every three days, and flow cytometry analysis on day 7 confirmed that the majority of bone marrow cells had differentiated into BMDCs.

### 2.10 Co-culture of CD4^+^ T cells with DCs

DCs were harvested from mouse bone marrow, while CD4^+^ T cells were isolated from mouse spleen. DCs were treated with APS for 2h and then treated with LPS for 48h, and CD4^+^ T cells were activated with anti-CD3 and anti-CD28 for 72h. Both cells were then co-cultured at a ratio of 5:1 (DCs: CD4^+^ T cells) for 72h (25).

### 2.11 Flow cytometry

For B16-OVA cells or DCs detection, following a 2-hour exposure to APS (200μg/mL), B16-OVA melanoma cells received interferon-γ (IFN-γ) (Peprotech) stimulation, while DCs were LPS-induced. Subsequently, both cells were harvested and stained with MHCII, CD80, CD86 and CD11c antibodies. For activated CD4^+^T cells detection, cells in DCs/CD4^+^T cells co-culture system, were stained with CD4 and CD69 antibodies. Cell sorting was performed utilizing the BD FACSLyric system, and subsequent data analysis was conducted with the FlowJo software (v10.8.1). The gating strategy for flow cytometry is presented in **Supplementary Figure 1**.

### 2.12 Statistical analysis

Statistical evaluations and quantitative analysis were conducted using GraphPad Prism 8 software and ImageJ software (Version 1.54). Data are expressed as mean± standard deviation (SD). Comparisons between two groups were performed using Student’s t-test, while comparisons among multiple groups were analyzed through one-way ANOVA followed by Tukey’s *post hoc* test. Unless otherwise specified, n represents independent biological replicates. Differences in tumor volume were assessed using two-way ANOVA with subsequent Tukey’s test. A P-value of less than 0.05 was regarded as statistically significant (*p < 0.05, **p < 0.01, ***p < 0.001, ****p < 0.0001).

## 3 Results

### 3.1 Network pharmacology predicted JAK/STAT1 signaling and TLR signaling as candidate pathways potentially involved in the effects of APS on anti PD-1 therapy

To predict whether APS can influence the therapeutic response of anti PD-1 to melanoma, we first performed a network pharmacology analysis before experimental validation. By intersecting potential targets of APS, melanoma-related genes, and anti PD-1 related targets, we identified 56 overlapping genes in melanoma potentially affected by APS and anti PD-1 (**Figure 1A**). A PPI network was constructed to visualize the relationships among these targets, revealing core nodes such as IL-6, TNF, and TLR4 (**Figure 1B**). GO enrichment analysis indicated that these targets were predominantly involved in biological processes such as cellular response to lipopolysaccharide (LPS) and cytokine activity (**Figure 1C**). Furthermore, KEGG pathway enrichment analysis highlighted that the JAK-STAT signaling and the TLR signaling were the top enriched signaling cascades (**Figure 1D**). Given accumulating evidences that JAK–STAT and TLR signaling could potentiate antitumor immunity and enhance the efficacy of anti PD-1 (26–29), we proposed that APS might modulate the therapeutic efficacy of anti PD-1 through JAK–STAT and TLR signaling.

**Figure 1:**
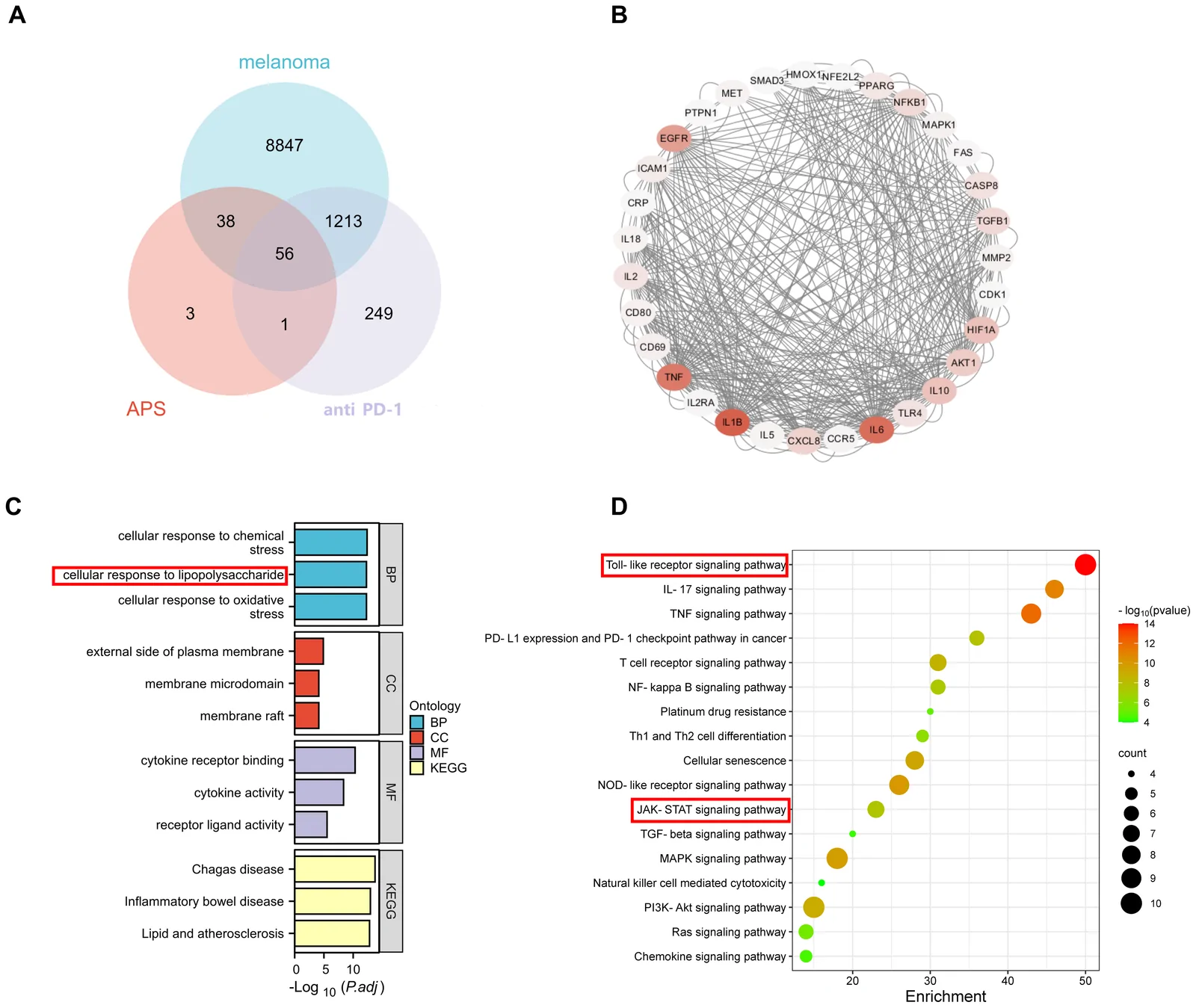
Network pharmacology predicted that APS significantly regulated TLR signaling and JAK/STAT signaling **(A)** Venn diagram of common targets among anti PD-1, APS and melanoma. **(B)** Protein-Protein Interaction Networks (PPI) showing the nodes (proteins) and edges (relationships between proteins). **(C)** Top 12 highly enriched GO terms associated with the target genes. BP, biological process; MF, molecular function; CC, cell component. **(D)** Top 17 enriched KEGG pathways associated with the target genes. APS, astragalus polysaccharide.

### 3.2 APS antagonized anti PD-1 efficacy in melanoma bearing mice

To systematically evaluate the therapeutic effects of APS in combination with anti PD-1 *in vivo*, we established a B16-OVA melanoma-bearing C57BL/6J mouse model. The intervention was initiated on the 8th day post-tumor inoculation, as illustrated in the experimental schematic (**Figure 2A**). APS alone group showed no difference in tumor volumes compared with the control group (**Figure 2B**). As anticipated, the administration of anti PD-1 alone significantly inhibited tumor growth compared to the control group (**Figure 2B**). However, APS combined with anti PD-1 group resulted in markedly increased tumor volumes compared to anti PD-1 alone group (**Figure 2B**). Individual tumor growth curves for each group further corroborated this trend (**Figures 2C–F**).

**Figure 2:**
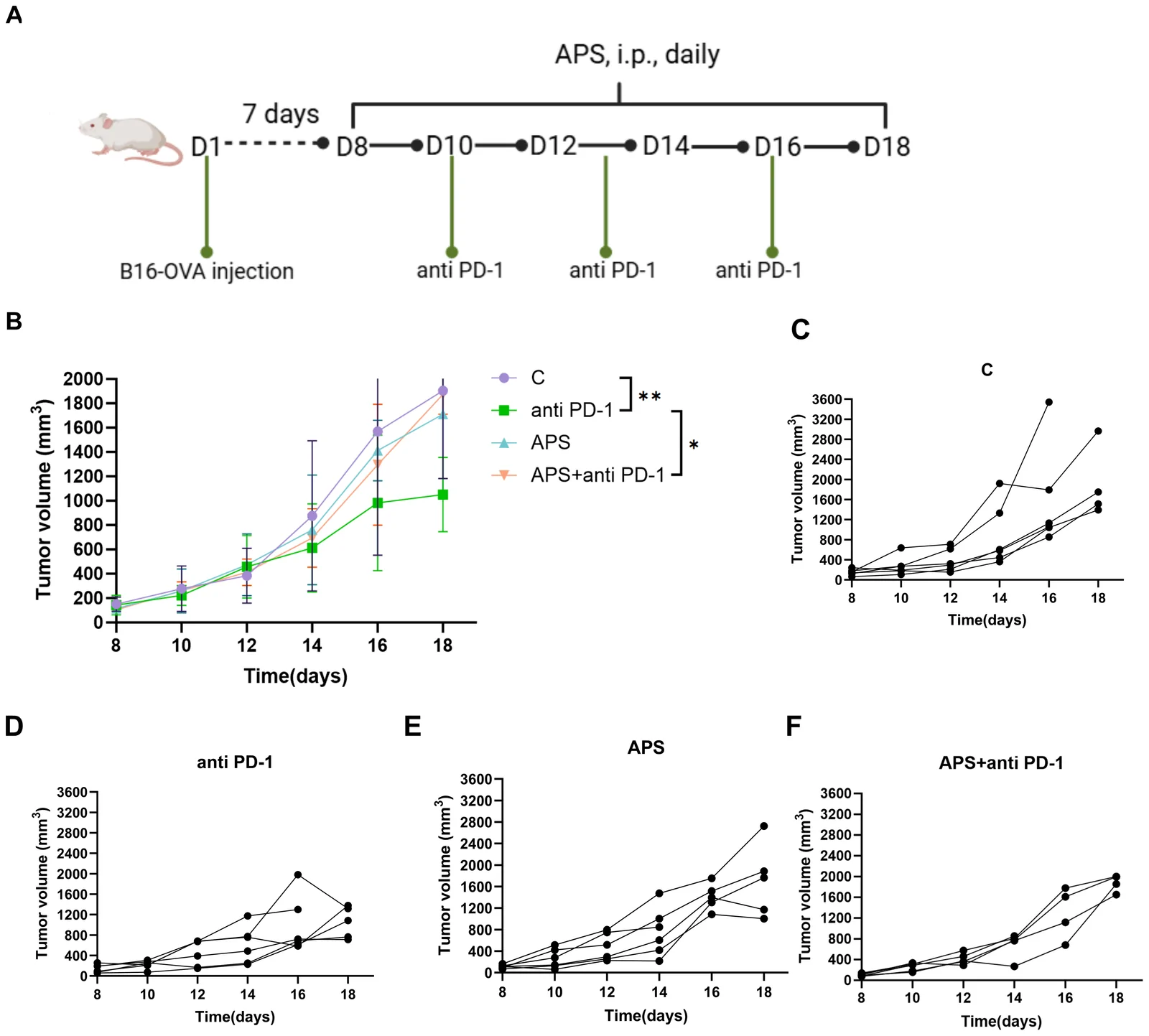
APS antagonized anti PD-1 efficacy in melanoma bearing mice. **(A)** Schematic illustration of mice experiment. **(B)** Tumor growth curves of B16-OVA bearing mice (n=6). **(C–F)** Individual tumor growth curve in Control **(C)**, anti PD-1 **(D)**, APS **(E)**, APS+anti PD-1 **(F)**, respectively (n=6). *p < 0.05, **p < 0.01. C, control; D, day; APS, Astragalus Polysaccharide; i.p., intraperitoneal.

### 3.3 APS reversed anti PD-1-induced immune cells infiltration in tumor microenvironment

To elucidate the immunity basis of the antagonistic effect of APS on anti PD-1, we performed IHC analysis to assess the infiltration of immune cells in the tumor tissues. The recruitment of T cells and DCs are critical for effective anti-tumor immune response. As expected, our results revealed that anti PD-1 alone group significantly promoted the infiltration of CD4^+^ T cells, CD8^+^ T cells, and CD11c^+^ DCs into the melanoma tissue, while these effects were markedly reversed by the addition of APS (**Figures 3A–C**).

**Figure 3:**
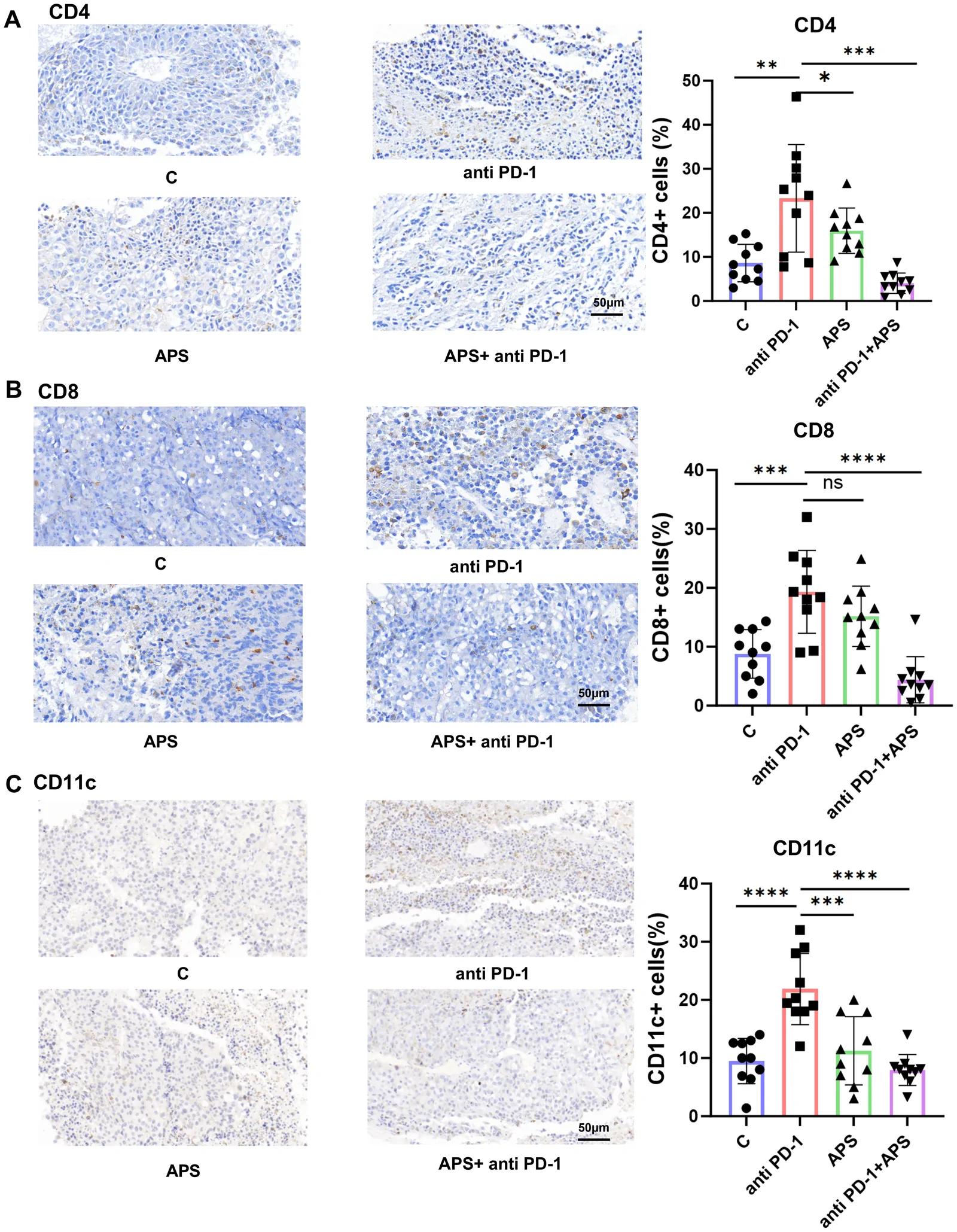
APS reduced the infiltration of immune cells in tumor microenvironment. **(A–C)** Left) Representative images of CD4 **(A)**, CD8 **(B)** and CD11c **(C)** immunohistochemistry in tumor of B16-OVA bearing mice (n=3) (40×magnification). **(A–C)** Right) Statistical data of CD4 **(A)**, CD8 **(B)** and CD11c **(C)** immunohistochemistry in tumor of B16-OVA bearing mice. *p < 0.05, **p < 0.01, ***p < 0.001, ****p < 0.0001. C, control; APS, astragalus polysaccharide.

### 3.4 APS inhibited the JAK-STAT1/CIITA signaling and downregulated MHCII expression

Since our initial network pharmacology analysis predicted JAK–STAT signaling as a candidate pathway, we next investigated whether APS could affect the JAK–STAT1 signaling. B16-OVA is a classic immunocompetent melanoma model stably expressing ovalbumin (OVA) as a defined tumor neoantigen, which is extensively applied to dissect MHC-II-dependent antigen presentation and anti PD-1 therapeutic efficacy in preclinical immunotherapy research (30). Meanwhile, DCs are professional antigen-presenting cells dominating MHC-II-mediated CD4^+^ T cell activation within the tumor immune microenvironment (31). It has been documented that MHC-II expression is strongly correlated with therapeutic efficacy and clinical prognosis in patients receiving anti PD-1 monotherapy or combination regimens (7). CIITA acts as the master transcriptional regulator governing MHC-II transcription, whose expression is tightly controlled by the canonical JAK-STAT1 signaling cascade (32). Our prior network pharmacology prediction indicated that APS exerts regulatory effects on the JAK-STAT1 signaling axis. We further validated the impact of APS on the JAK-STAT1 signaling by western blot. Western blot results revealed that APS treatment significantly inhibited the phosphorylation of STAT1 (P-STAT1) in B16-OVA cells (**Figures 4A, C**) and DCs (**Figures 4D, F**). Our data also showed that APS treatment could down-regulate the mRNA level of *Ciita* and protein level of CIITA in B16-OVA cells and DCs (**Figures 4B, C, E, F**).

**Figure 4:**
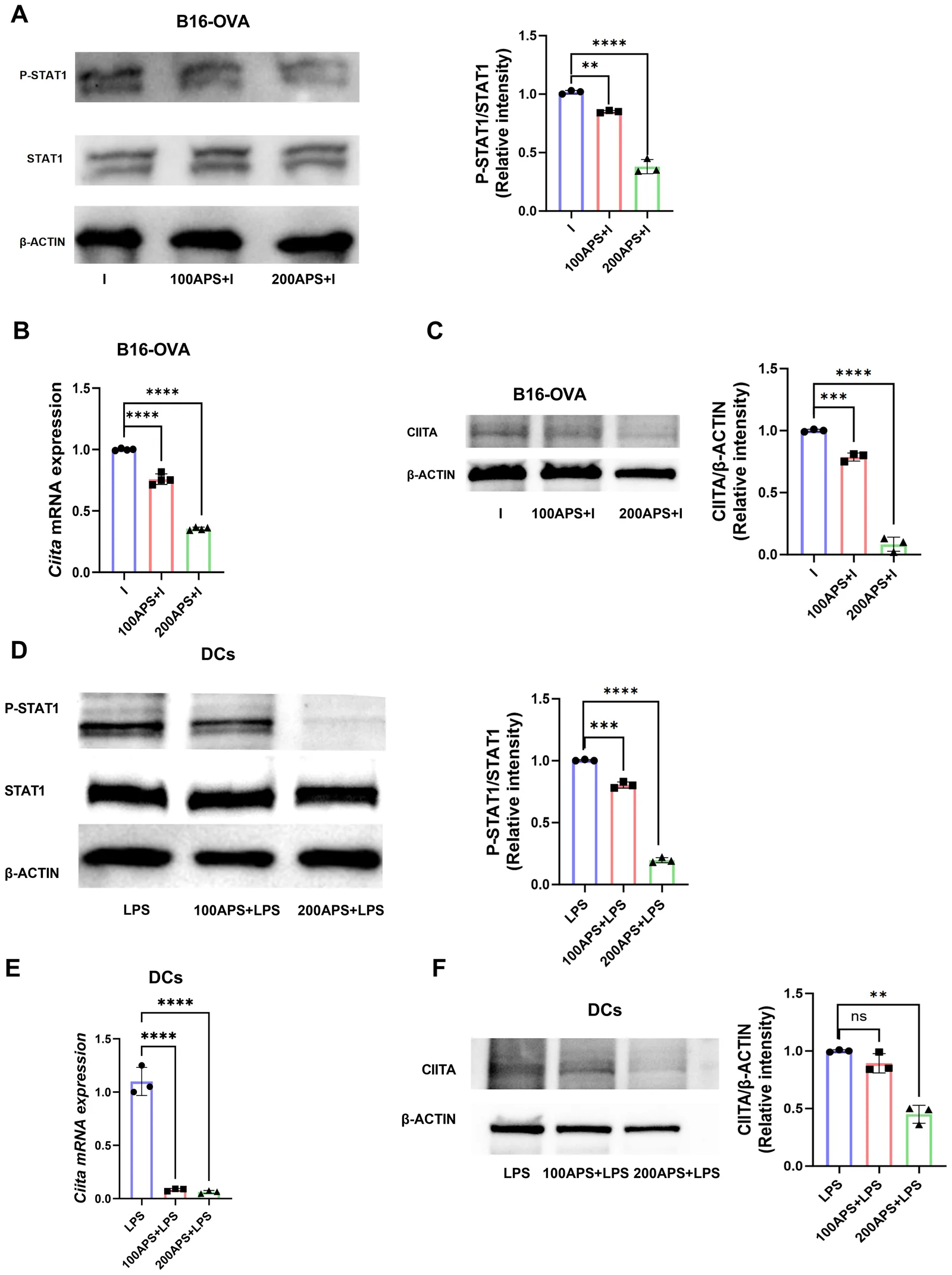
APS inhibited JAK/STAT1 signaling and downregulated CIITA expression in B16-OVA cells and DCs. **(A, C)** Representative images of Western blots and quantification of P-STAT1, STAT1 **(A)** and CIITA **(C)** in B16-OVA cells. **(B)** Relative mRNA expression of *Ciita* in B16-OVA cells. **(D, F)** Representative images of Western blots and quantification of P-STAT1, STAT1 **(D)** and CIITA **(F)** in DCs. **(E)** Relative mRNA expression of *Ciita* in DCs. **p < 0.01, ***p < 0.001, ****p < 0.0001; ns, not significant. APS, astragalus polysaccharide (100APS: 100μg/ml; 200APS: 200μg/ml); I, interferon γ; LPS, lipopolysaccharide; DCs, dendritic cells; CIITA, class II major histocompatibility complex transactivator; STAT1, signal transducer and activator of transcription 1; p-STAT1, phosphorylated signal transducer and activator of transcription 1.

Consistent with the suppression of CIITA, APS treatment led to a significant down-regulation of MHCII molecules. We observed that APS treatment markedly reduced mRNA levels of MHCII subunits (*H2Aa*, *H2Ab1*) in both B16-OVA cells (**Figure 5A**) and DCs (**Figure 5C**). The flow cytometry data also demonstrated that APS treatment significantly reduced the expression of MHCII in B16-OVA cells and DCs (**Figures 5B, D**). IHC data revealed that anti PD-1 alone promoted the MHCII expression in the melanoma tissue, while these effects were markedly reversed by the addition of APS (**Figure 5E**).

**Figure 5:**
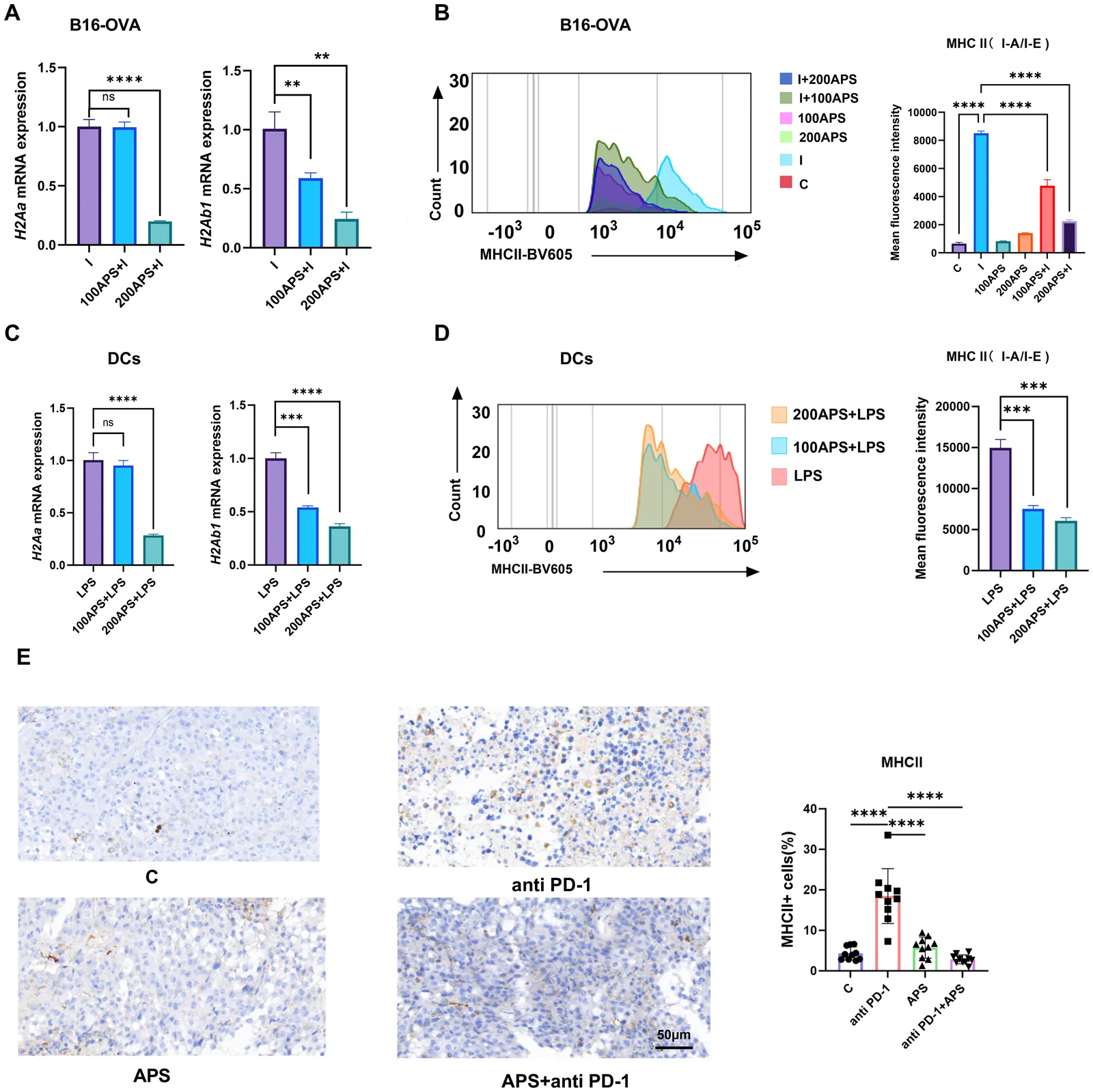
APS downregulated MHCII expression in B16-OVA Cells and DCs. Relative mRNA expressions of MHCII genes (*H2Aa*, *H2Ab1*) in B16-OVA cells **(A)** and DCs **(C)**. Representative flow cytometry images and mean fluorescence intensity (MFI) of MHCII in B16-OVA cells **(B)** and DCs **(D)**. **(E)** Representative immunohistochemistry images and statistical analysis of MHCII in B16-OVA melanoma tissues (n=3). **p < 0.01, ***p < 0.001, ****p < 0.0001; ns, not significant. APS, astragalus polysaccharide (100APS: 100μg/ml; 200APS: 200μg/ml); C, control; I, interferon γ; LPS, lipopolysaccharide; DCs, dendritic cells; MHCII, major histocompatibility complex class II.

### 3.5 APS downregulated TLR/NF-κB signaling and suppressed pro-inflammatory cytokines

Aberrant activation of the TLR-NF-κB cascade within the melanoma microenvironment fuels chronic inflammatory responses, and accumulating evidence has verified that TLR pathway activation improves the therapeutic responsiveness of tumor lesions to anti PD-1 immunotherapy (33). Guided by the network pharmacology predictions, we validated the impact of APS on the TLR signaling using PCR array analyses in B16-OVA cells. Volcano plot visualization displayed globally divergent transcriptional signatures between the control group and APS-treated group (**Figure 6A**). Quantitative analysis of PCR array data further validated that APS exposure significantly repressed the transcription of core TLR signaling mediators, encompassing upstream damage-associated molecular pattern gene *Hmgb1*, intermediate signaling molecules *Hras1* and *Map2k4*, interferon gene *Ifnb1*, as well as pattern recognition receptors *Tlr2*, *Tlr3* and *Tlr6* (**Figures 6B–H**).

**Figure 6:**
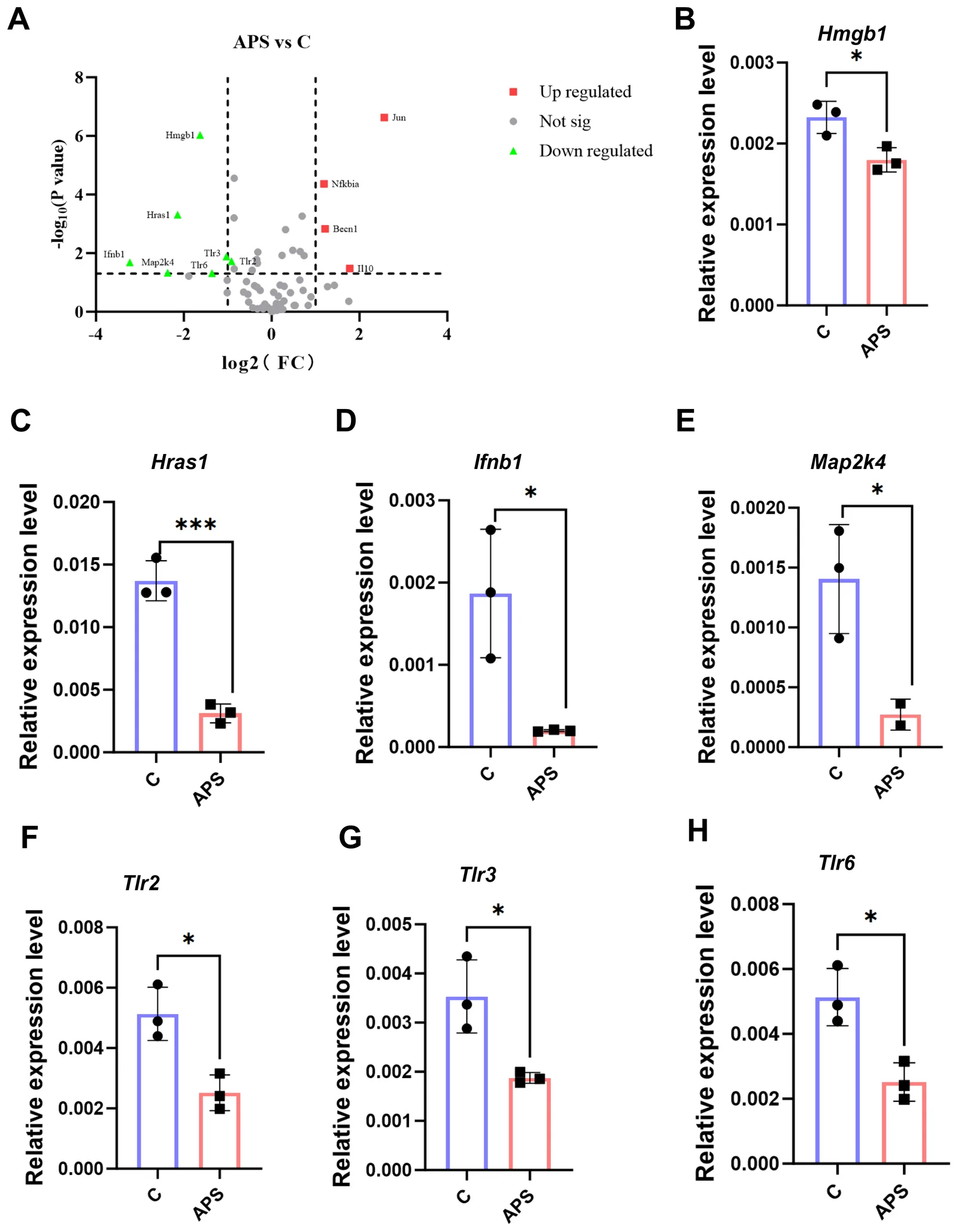
APS downregulated Toll-like receptor signaling pathway. **(A)** Volcano plot of PCR array for Toll-like receptor signaling (n=3). **(B–H)** Downregulated genes in Toll-like receptor signaling, including *Hmgb1* **(B)**, *Hras1* **(C)**, *Ifnb1* **(D)**, *Map2k4* **(E)**, *Tlr2* **(F)**, *Tlr3* **(G)**, and *Tlr6* **(H)**. *p < 0.05, ***p < 0.001. APS, Astragalus Polysaccharide; C, control; *Hmgb1*, High Mobility Group Box 1; *Hras1*, Harvey Rat Sarcoma Viral Oncogene Homolog 1; *Ifnb1*, Interferon Induced with Helicase C Domain 1; *Map2k4*, mitogen-activated protein kinase kinase 4; *Tlr2/3/6*, toll like receptor 2/3/6.

TLR signal transduction canonically propagates downstream NF-κB nuclear translocation to initiate robust pro-inflammatory transcriptional programs. On the basis of the above transcriptional screening results, we performed immunofluorescence staining to detect the subcellular localization of the NF-κB p65 subunit. It revealed that APS treatment markedly inhibited the LPS/IFNγ - induced nuclear accumulation of NF-κB in B16-OVA cells (**Figures 7A, B**). Consequently, the production of key pro-inflammatory cytokines (TNF-α and IL-6) in B16-OVA cells (**Figures 7C–F**) and DCs (**Figures 7G–J**), were also suppressed by APS treatment.

**Figure 7:**
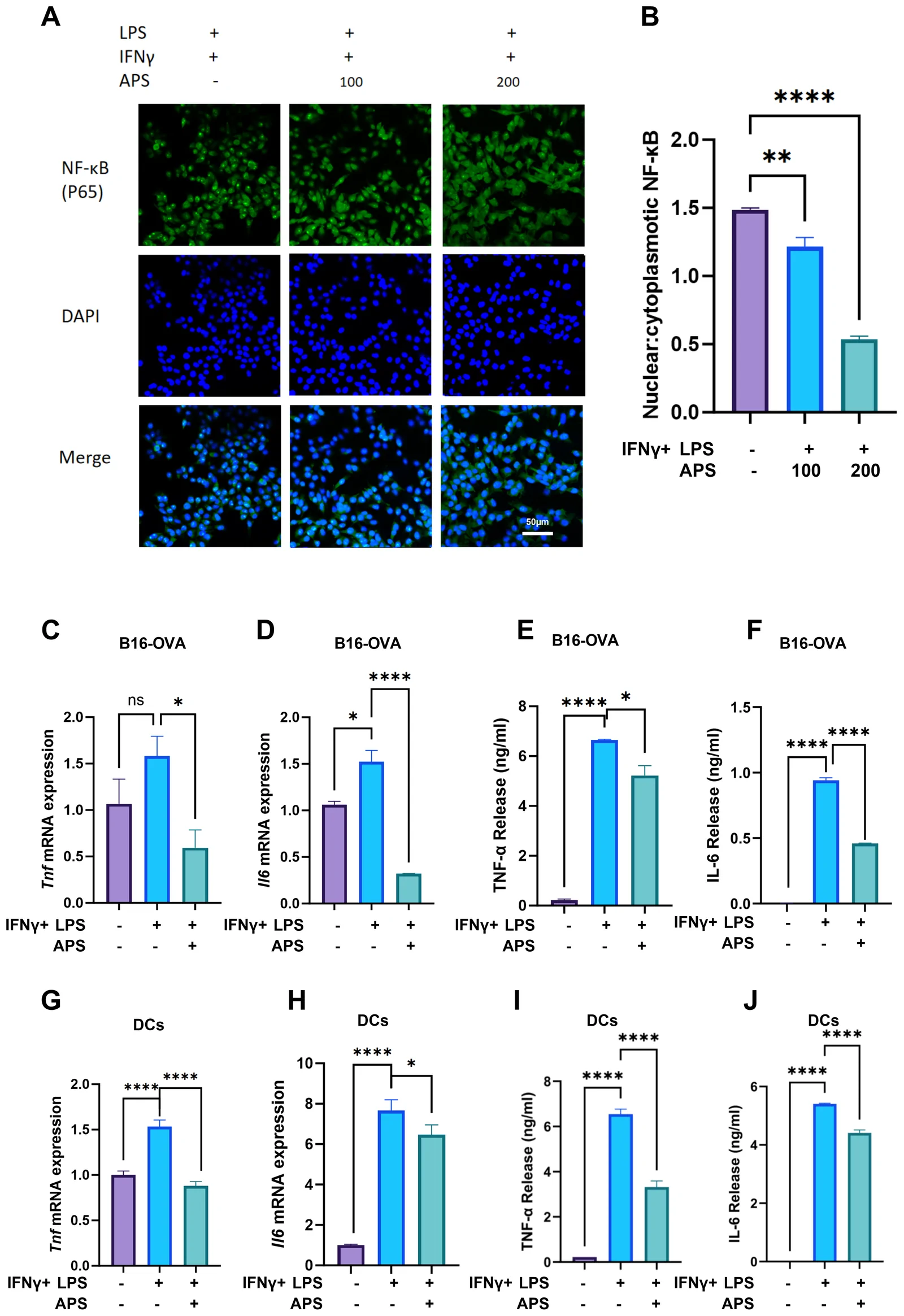
APS inactivated NF-κB signaling and suppressed pro-inflammatory cytokines secretion. **(A)** Representative immunofluorescence images of NF-κB (p65) in B16-OVA cells. **(B)** Quantification of the nuclear/cytoplasmic fluorescence intensity ratio of NF-κB (n=3). Relative mRNA expression **(C, D)** of *Tnf* and *Il6*, and protein levels **(E, F)** of TNF-α and IL-6 in B16-OVA cells. Relative mRNA expression **(G, H)** of *Tnf* and *Il6* and protein levels **(I, J)** of TNF-α and IL-6 in DCs. *p < 0.05, **p < 0.01, ****p < 0.0001. APS, Astragalus Polysaccharide (100APS: 100μg/ml; 200APS: 200μg/ml); C, control; LPS, lipopolysaccharide; DCs, dendritic cells; NF-κB, nuclear factor kappa-B; IFN-γ, interferon gamma; TNF-α, tumor necrosis factor alpha; IL-6, interleukin 6.

### 3.6 APS impaired DC maturation and subsequent CD4^+^ T cell activation

Combined with the above molecular findings, APS interferes with two pivotal immunoregulatory axes in DCs and melanoma cells: the JAK-STAT1/CIITA/MHC-II antigen presentation pathway and the TLR/NF-κB pro-inflammatory signaling cascade. Since mature DCs rely on intact MHC-II antigen presentation and inflammatory costimulatory signals to drive effective CD4^+^ T cell antitumor immunity (34), we next characterized the functional phenotypes of DCs upon APS administration. To determine the functional consequences of APS treatment on adaptive immunity, we evaluated the maturation status of DCs and their ability to activate CD4^+^T cells after APS treatment. Flow cytometry analysis revealed that APS treatment significantly downregulated the expression of key DC maturation markers, including MHC-II, CD11c, CD80, and CD86 (**Figures 5D**, **8A–D**). We further established a DCs and CD4^+^T cells co-culture system (**Figure 8E**), and found that DCs treated with APS exhibited a reduced capacity to activate CD4^+^ T cells. This was evidenced by the significantly decreased CD69 (early activation marker) in CD4^+^ T cells (**Figure 8F**). Furthermore, ELISA analysis of the co-culture supernatant demonstrated that APS treatment markedly suppressed the release of CD4^+^T cell effector cytokines, specifically TNF-α and IFN-γ (**Figures 8G, H**).

**Figure 8:**
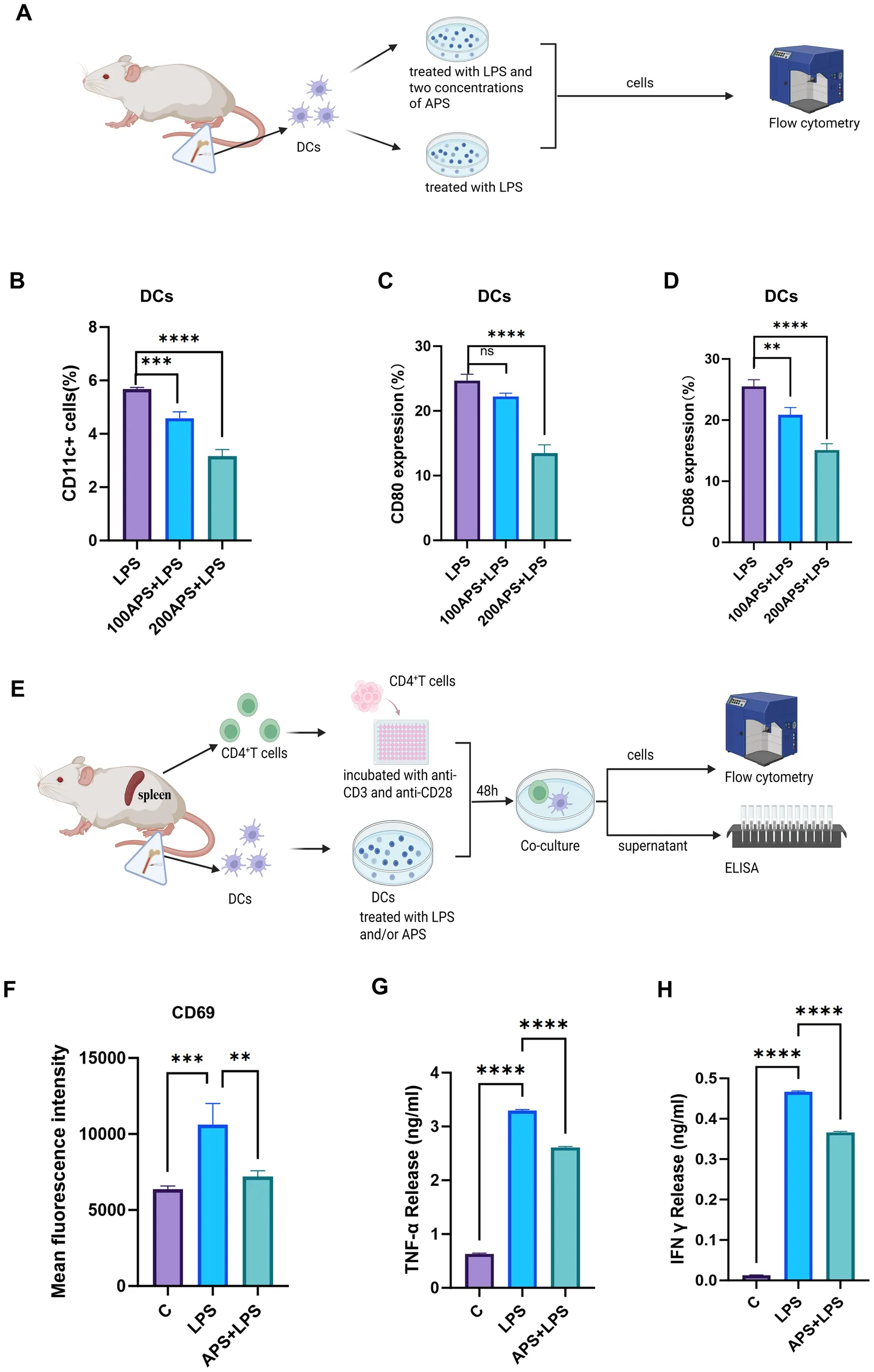
APS impaired DCs maturation and inhibited CD4^+^T cell activation in the DCs and CD4^+^T cell co-culture system. **(A)** Schematic illustration of experimental procedures for detecting DC maturation upon APS intervention. Percentage of CD11c^+^ **(B)**, CD80^+^ **(C)**, and CD86^+^ **(D)** cells in DCs determined by flow cytometry (n=3). **(E)** Schematic illustration of the DCs and CD4^+^ T cell co-culture system. **(F)** Percentage of CD69^+^ cells in CD4^+^T cells determined by flow cytometry (n=3). Concentration of TNF-α **(G)** and IFN-γ **(H)** in the co-culture supernatant determined by ELISA (n=3). **p<0.01, ***p<0.001, ****p<0.0001, ns, not significant. APS, Astragalus Polysaccharide (100APS, 100μg/ml; 200APS, 200μg/ml); DCs, dendritic cells; C, control; LPS, Lipopolysaccharide; IFN-γ, interferon gamma; TNF-α, tumor necrosis factor alpha.

## 4 Discussion

Anti PD-1 has revolutionized the treatment landscape for advanced melanoma (3). However, resistance to anti PD-1 remains a major clinical challenge (3). Seeking PD-1 sensitizers from phytomedicines represents a vital research direction (35–37). Our previous study reported that *Astragalus* injection exerted an antagonistic effect on anti PD-1 treatment (24). It has been reported that the primary active component of *Astragalus* is astragalosides, whereas the content of APS in *Astragalus* injection is very low (38). APS itself is a recognized immunomodulatory drug (16), nevertheless, its regulatory effect on anti PD-1 treatment efficacy is still unclarified. In this study, we evaluated the efficacy of APS combined with anti PD-1 on melanoma and found that APS markedly impaired anti PD-1 efficacy in melanoma. Polysaccharides are widely distributed in medical plants (39), our previous experimental results also indicated that lentinan, another medicinal polysaccharide extracted from *Lentinus edodes*, exhibited a similar antagonistic effect on anti PD-1 efficacy (data not published). These findings suggest that natural polysaccharides might inherently exert antagonistic effects against immune checkpoint inhibitors.

Immune cells infiltration in tumor microenvironment is highly correlated with the therapeutic efficacy of anti PD-1 (40). In this study, we found that APS induced an immunosuppressive microenvironment, which was evidenced by reduced infiltration of CD4^+^ T cells, CD8^+^ T cells, and CD11c^+^ DCs in APS and anti PD-1 treated melanoma. We also observed that APS impaired DCs maturation, and subsequent CD4^+^T cell activation in anti PD-1 treated melanoma. These findings provide a potential immunological explanation for the reduced efficacy of anti PD-1 therapy. In addition, although IHC analysis demonstrated that APS reversed the anti PD-1 induced increase in CD8^+^ T-cell infiltration, the functional status of tumor-infiltrating CD8^+^ T cells were not directly assessed. In particular, cytotoxic effector molecules such as granzyme B, perforin, and IFN-γ in CD8^+^ T cells were not evaluated. Besides, although DC maturation was evaluated based on the expression of CD11c, CD80, CD86, and MHC-II, additional markers associated with DC maturation, such as CD40 and CCR7 (41), were not examined.

MHCII molecules are essential for antigen presentation to CD4^+^ T cells (11). Increasing evidences indicate that both APC-derived MHCII and tsMHCII are associated with tumor immunogenicity (7, 11). MHCII molecules show strong correlation with treatment response and prognosis in patients receiving anti PD-1 (43). In this study, APS markedly reduced MHCII expression in both DCs and melanoma cells, suggesting impaired antigen presentation capacity within the tumor microenvironment. MHCII transcription is governed by CIITA, which acts as its master regulator (11). CIITA itself is tightly controlled by the JAK-STAT pathway (12). Our data showed that APS significantly inhibited STAT1 phosphorylation and reduced both *Ciita* mRNA and CIITA protein expression. In addition, upstream JAK1/JAK2 phosphorylation and contributions from other STAT family members were not directly assessed. Besides the JAK–STAT1 pathway, CIITA is also subject to regulation by other factors, including IRF1, BRG1, and FBXO11 (44), which were also not determined in this study.

Activation of TLR pathway is known to sensitize tumors to anti PD-1 therapy (33). Reduced TLR/NF-κB signaling could diminish inflammatory cytokine production and DC costimulatory activity, thereby creating conditions less favorable for effective T-cell priming (45). Our *in vitro* data indicated that APS markedly suppressed TLR signaling and attenuated NF-κB mediated inflammatory responses in B16-OVA melanoma cells and DCs. This finding provided another potential mechanism that APS exerted antagonistic effects against anti PD-1. However, our study only evaluated NF-κB p65 nuclear translocation and TNF-α/IL-6 production *in vitro*. These findings should be further validated in tumor tissues.

In addition, other limitations of this work should be acknowledged. First, only a single dose of APS was evaluated *in vivo* without dose-gradient experiments, leaving the dose–response relationship of APS-mediated antagonism of anti PD-1 therapy undetermined. Second, *in vivo* findings were derived from a single B16-OVA melanoma model with a modest cohort size. The single tumor model and limited cohort may still restrict the generalizability of results across other tumor contexts. Third, mechanistic experiments were predominantly conducted using murine cells and tumor tissues, with no validation in human samples; caution is thus warranted when extrapolating findings to patients. Fourth, major immunosuppressive cell populations in the tumor microenvironment, including Tregs, MDSCs and M2-polarized macrophages, were not systematically profiled, and their potential contributions to the observed APS-associated phenotype cannot be excluded. Fifth, the standard B16-OVA melanoma model in healthy young mice did not fully recapitulate the severe frailty, impaired hepatic/renal function and immune exhaustion typical of advanced cancer patients, who constitute the primary population receiving APS in clinical practice.

## 5 Conclusion

APS markedly antagonized the therapeutic efficacy of anti PD-1 in melanoma by inducing an immunosuppressive microenvironment. These effects were at least partially dependent on inhibition of JAK-STAT1/CIITA and TLR/NF-κB signaling. To our knowledge, this is the first report that natural polysaccharides could antagonize the efficacy of ICIs in cancer. This study suggested that natural polysaccharides combined with ICIs should be used with caution for cancer patients.

## Data Availability

The original contributions presented in the study are included in the article/**Supplementary Material**. Further inquiries can be directed to the corresponding authors.

## References

[1] Davis LE Shalin SC Tackett AJ . Current state of melanoma diagnosis and treatment. Cancer Biol Ther. (2019) 20:1366–79. doi: 10.1080/15384047.2019.1640032 31366280 PMC6804807

[2] Wang X Ma S Zhu S Zhu L Guo W . Advances in immunotherapy and targeted therapy of Malignant melanoma. Biomedicines. (2025) 13:225. doi: 10.3390/biomedicines13010225 39857808 PMC11761959

[3] Kleffel S Posch C Barthel SR Mueller H Schlapbach C Guenova E . Melanoma cell-intrinsic PD-1 receptor functions promote tumor growth. Cell. (2015) 162:1242–56. doi: 10.1016/j.cell.2015.08.052 26359984 PMC4700833

[4] Topchyan P Lin S Cui W . The role of CD4 T cell help in CD8 T cell differentiation and function during chronic infection and cancer. Immune Netw. (2023) 23:e41. doi: 10.4110/in.2023.23.e41 37970230 PMC10643329

[5] Marzagalli M Ebelt ND Manuel ER . Unraveling the crosstalk between melanoma and immune cells in the tumor microenvironment. Semin Cancer Biol. (2019) 59:236–50. doi: 10.1016/j.semcancer.2019.08.002 31404607

[6] Maccy AM Herrmann LM Adams AC Hastings KT . Major histocompatibility complex class II in the tumor microenvironment: functions of nonprofessional antigen-presenting cells. Curr Opin Immunol. (2023) 83:102330. doi: 10.1016/j.coi.2023.102330 37130456 PMC10524529

[7] Johnson DB Estrada MV Salgado R Sanchez V Doxie DB Opalenik SR . Melanoma-specific MHC-II expression represents a tumour-autonomous phenotype and predicts response to anti-PD-1/PD-L1 therapy. Nat Commun. (2016) 7:10582. doi: 10.1038/ncomms10582 26822383 PMC4740184

[8] Reith W LeibundGut-Landmann S Waldburger J-M . Regulation of MHC class II gene expression by the class II transactiva tor. Nat Rev Immunol. (2005) 5:793–806. doi: 10.1016/s0171-2985(11)80550-4 16200082

[9] Muhlethaler-Mottet A Di Berardino W Otten LA Mach B . Activation of the MHC class II transactivator CIITA by interferon-γ requires cooperative interaction between Stat1 and USF-1. Immunity. (1998) 8:157–66. doi: 10.1016/s1074-7613(00)80468-9 9491997

[10] Holzgruber J Martins C Kulcsar Z Duplaine A Rasbach E Migayron L . Type I interferon signaling induces melanoma cell-intrinsic PD-1 and its inhibition antagonizes immune checkpoint blockade. Nat Commun. (2024) 15:7165. doi: 10.1038/s41467-024-51496-2 39187481 PMC11347607

[11] Couture A Gagnier A Décazes F Boyer F de Guibert J Le Fourn V . HLA-class II artificial antigen presenting cells in CD4+ T cell-based immunotherapy. Front Immunol. (2019) 10. doi: 10.3389/fimmu.2019.01081 31156634 PMC6533590

[12] Duan T Du Y Xing C Wang HY Wang R . Toll-like receptor signaling and its role in cell-mediated immunity. Front Immunol. (2022) 13:812774. doi: 10.3389/fimmu.2022.812774 35309296 PMC8927970

[13] Chen X Zhang Y Fu Y . The critical role of toll-like receptor-mediated signaling in cancer immunotherapy. Med Drug Discov. (2022) 14:100122. doi: 10.1016/j.medidd.2022.10012242574925

[14] Xiong H Han X Cai L Zheng H . Natural polysaccharides exert anti-tumor effects as dendritic cell immune enhancers. Front Oncol. (2023) 13:1274048. doi: 10.3389/fonc.2023.1274048 37876967 PMC10593453

[15] Chen Y Li H Lai F Min T Wu H Zhan Q . The influence and mechanisms of natural plant polysaccharides on intestinal microbiota-mediated metabolic disorders. Foods. (2024) 13:3882. doi: 10.3390/foods13233882 39682954 PMC11640612

[16] Chen J-P Lai H-C . Immuno-potentiating effects of *Astragalus* polysaccharides: a mini-literature review. J Cancer Res Pract. (2020) 7:99. doi: 10.4103/jcrp.jcrp_3_2042658757

[17] Yu H Ding G Gong Q Ma J Zhao Y Wang Y . Modulation of PD-L1 by Astragalus polysaccharide attenuates the induction of melanoma stem cell properties and overcomes immune evasion. BMC Cancer. (2024) 24:1034. doi: 10.1186/s12885-024-12788-4 39169294 PMC11340040

[18] Liu X Xia W Wei J Liu Y Li M Yu J . Therapeutic effect of Astragalus polysaccharides on hepatocellular carcinoma H22-bearing mice. Dose Response. (2017) 15:1559325816685182. doi: 10.1177/1559325816685182 28210201 PMC5298564

[19] Tian QE Li HD Yan M Cai HL Tan QY Zhang WY . Astragalus polysaccharides can regulate cytokine and P-glycoprotein expression in H22 tumor-bearing mice. World J Gastroenterol. (2012) 18:7079–86. doi: 10.3748/wjg.v18.i47.7079 23323011 PMC3531697

[20] Pangiao O Morrison RM Chauvin J-M Algazi A Daud A Tsai K . Tim-3 mediates T cell trogocytosis to limit antitumor immunity. J Clin Invest. 132(9):e152864. doi: 10.1172/jci152864 35316223 PMC9057587

[21] Gong J Le TQ Massarelli E Hendifar AE Tuli R . Radiation therapy and PD-1/PD-L1 blockade: the clinical development of an evolving anticancer combination. J Immunother Cancer. (2018) 6:46. doi: 10.1186/s40425-018-0361-7 29866197 PMC5987486

[22] Fox DB Bright RY Hong X Russell HC Guo H LaSalle TJ . Downregulation of KEAP1 in melanoma promotes resistance to immune checkpoint blockade. NPJ Precis Oncol. (2023) 7:25. doi: 10.1038/s41698-023-00362-3 36864091 PMC9981575

[23] Peng S Yan Y Ngai T Li J Ogino K Xia Y . Development and optimal immune strategy of an alum-stabilized Pickering emulsion for cancer vaccines. Vaccines. (2023) 11:1169. doi: 10.3390/vaccines11071169 37514985 PMC10383433

[24] Lei R Li Q Wang R Wu Z Kong Q Liu S . Astragalus injection antagonizes the efficacy of anti-PD-1 against melanoma through down-regulating MHC-II expression. J Ethnopharmacol. (2025) 349:119966. doi: 10.1016/j.jep.2025.119966 40354837

[25] Malik-Chaudhry HK Prabhakar K Ugamraj HS Boudreau AA Buelow B Dang K . TNB-486 induces potent tumor cell cytotoxicity coupled with low cytokine release in preclinical models of B-NHL. mAbs. 13(1):1890411. doi: 10.1080/19420862.2021.1890411 33818299 PMC8023237

[26] Sato-Kaneko F Yao S Ahmadi A Zhang SS Hosoya T Kaneda MM . Combination immunotherapy with TLR agonists and checkpoint inhibitors suppresses head and neck cancer. JCI Insight. (2017) 2. doi: 10.1172/jci.insight.93397 28931759 PMC5621908

[27] Ribas A Medina T Kummar S Amin A Kalbasi A Drabick JJ . SD-101 in combination with pembrolizumab in advanced melanoma: results of a phase Ib, multicenter study. Cancer Discov. (2018) 8:1250–7. doi: 10.1158/2159-8290.cd-18-0280 30154193 PMC6719557

[28] Zaretsky JM Garcia-Diaz A Shin DS Escuin-Ordinas H Hugo W Hu-Lieskovan S . Mutations associated with acquired resistance to PD-1 blockade in melanoma. N Engl J Med. (2016) 375:819–29. doi: 10.1056/nejmoa1604958 27433843 PMC5007206

[29] Garcia-Diaz A Shin DS Moreno BH Saco J Escuin-Ordinas H Rodriguez GA . Interferon receptor signaling pathways regulating PD-L1 and PD-L2 expression. Cell Rep. (2017) 19:1189–201. doi: 10.1016/j.celrep.2017.04.031 28494868 PMC6420824

[30] Jeong YS Lee KJ Kim YJ Lee SJ Koom WS Lee IJ . Investigating tumor immunogenicity as a determinant of differential abscopal effects. J Radiat Res. (2025) 66:253–63. doi: 10.1093/jrr/rraf021 40302523 PMC12100475

[31] Bandola-Simon J Ito Y Wucherpfennig KW Roche PA . Defective removal of invariant chain peptides from MHC class II suppresses tumor antigen presentation and promotes tumor growth. Cell Rep. (2025) 44:115150. doi: 10.1016/j.celrep.2024.115150 39752250 PMC11886875

[32] Morris AC Beresford GW Mooney MR Boss JM . Kinetics of a gamma interferon response: expression and assembly of CIITA promoter IV and inhibition by methylation. Mol Cell Biol. (2002) 22:4781–91. doi: 10.1128/mcb.22.13.4781-4791.2002 12052885 PMC133907

[33] Lee J Im K Gil S Na H Min G Kim N . TLR5 agonist in combination with anti-PD-1 treatment enhances anti-tumor effect through M1/M2 macrophage polarization shift and CD8(+) T cell priming. Cancer Immunol Immunother. (2024) 73:102. doi: 10.1007/s00262-024-03679-5 38630304 PMC11024077

[34] Hilligan KL Ronchese F . Antigen presentation by dendritic cells and their instruction of CD4+ T helper cell responses. Cell Mol Immunol. (2020) 17:587–99. doi: 10.1038/s41423-020-0465-0 32433540 PMC7264306

[35] Chamcheu JC Ba Q Ma H . Editorial: Natural products modulate the sensitivity of cancer to anti-PD-1 based immunotherapy. In: Frontiers in Pharmacology. Lausanne: Frontiers Media SA (2023) 14:1232456. doi: 10.3389/fphar.2023.1232456 PMC10317057 37405053

[36] Zheng H Wang G Liu M Cheng H . Traditional Chinese medicine inhibits PD-1/PD-L1 axis to sensitize cancer immunotherapy: a literature review. Front Oncol. (2023) 13:2023. doi: 10.3389/fonc.2023.1168226 37397393 PMC10312112

[37] Kokolus KM Haley JS Koubek EJ Gowda R Dinavahi SS Sharma A . Schweinfurthin natural products induce regression of murine melanoma and pair with anti-PD-1 therapy to facilitate durable tumor immunity. Oncoimmunology. (2019) 8:e1539614. doi: 10.1080/2162402x.2018.1539614 30713799 PMC6343772

[38] Chen Y Jiang ZT Chen XF Yu H Ju WZ Li CY . Determination of polysaccharide content in Astragalus oral liquid and injection by phenol-sulfuric acid method. Res Pract Chin Medicines. (2015) 29:64–65.

[39] Fan X Li K Qin X Li Z Du Y . Advances in the preparation and bioactivity of polysaccharides from medicinal plants with different molecular weights: a review. Chem Biodivers. (2025) 22:e03031. doi: 10.1002/cbdv.202403031 40609025

[40] Ku BM Kim Y Lee KY Kim S Sun J Lee S . Tumor infiltrated immune cell types support distinct immune checkpoint inhibitor outcomes in patients with advanced non-small cell lung cancer. Eur J Immunol. (2021) 51:956–64. doi: 10.1002/eji.202048966 33506525 PMC8248238

[41] He T Tang C Xu S Moyana T Xiang J . Interferon gamma stimulates cellular maturation of dendritic cell line DC2.4 leading to induction of efficient cytotoxic T cell responses and antitumor immunity. Cell Mol Immunol. (2007) 4:105–111. 17484804

[43] Johnson AM Bullock BL Neuwelt AJ Poczobutt JM Kaspar RE Li HY . Cancer cell–intrinsic expression of MHC class II regulates the immune microenvironment and response to anti–PD-1 therapy in lung adenocarcinoma. J Immunol. (2020) 204:2295–307. doi: 10.4049/jimmunol.1900778 32179637 PMC7472648

[44] Hou J Chen SN Gan Z Li N Huang L Huo HJ . In primitive zebrafish, MHC class II expression is regulated by IFN-γ, IRF1, and two forms of CIITA. J Immunol. (2020) 204:2401–15. doi: 10.4049/jimmunol.1801480 32188757

[45] Idoyaga J Moreno J Bonifaz L . Tumor cells prevent mouse dendritic cell maturation induced by TLR ligands. Cancer Immunol Immunother. (2007) 56:1237–50. doi: 10.1007/s00262-006-0275-y 17237931 PMC11029892

