# Supplementary material for: Astragalus polysaccharide antagonized anti PD-1 efficacy in melanoma through inhibiting JAK-STAT1/CIITA and TLR/NF-κB signaling
Source: Front Immunol. 2026 Sep 14;17:1947786. doi: 10.3389/fimmu.2026.1947786 (PMC13616731; doi:10.3389/fimmu.2026.1947786)
Supplement: Supplementary file 1 [file SupplementaryFile1.docx]

Supplementary Information

Abstract graph

**Supplementary Figure1.** **Gating strategy in flow cytometry**

(**A**) Representative gating strategy for CD11c^+^, CD80^+^, and CD86^+^ in DCs. (**B**) Representative gating strategy for CD69^+^ in CD4^+^ T cells. DC: dendritic cell.
